# Detection of Low Back Physiotherapy Exercises With Inertial Sensors and Machine Learning: Algorithm Development and Validation

**DOI:** 10.2196/38689

**Published:** 2022-08-23

**Authors:** Abdalrahman Alfakir, Colin Arrowsmith, David Burns, Helen Razmjou, Michael Hardisty, Cari Whyne

**Affiliations:** 1 Holland Bone and Joint Program Sunnybrook Research Institute Toronto, ON Canada; 2 Institute of Biomedical Engineering University of Toronto Toronto, ON Canada; 3 Halterix Corporation Toronto, ON Canada; 4 Division of Orthopaedic Surgery Department of Surgery University of Toronto Toronto, ON Canada

**Keywords:** low back pain, rehabilitation, wearables, inertial measurement units, machine learning, activity recognition

## Abstract

**Background:**

Physiotherapy is a critical element in the successful conservative management of low back pain (LBP). A gold standard for quantitatively measuring physiotherapy participation is crucial to understanding physiotherapy adherence in managing recovery from LBP.

**Objective:**

This study aimed to develop and evaluate a system with wearable inertial sensors to objectively detect the performance of unsupervised exercises for LBP comprising movement in multiple planes and sitting postures.

**Methods:**

A quantitative classification design was used within a machine learning framework to detect exercise performance and posture in a cohort of healthy participants. A set of 8 inertial sensors were placed on the participants, and data were acquired as they performed 7 McKenzie low back exercises and 3 sitting posture positions. Engineered time series features were extracted from the data and used to train 9 models by using a 6-fold cross-validation approach, from which the best 2 models were selected for further study. In addition, a convolutional neural network was trained directly on the time series data. A feature importance analysis was performed to identify sensor locations and channels that contributed the most to the models. Finally, a subset of sensor locations and channels was included in a hyperparameter grid search to identify the optimal sensor configuration and best performing algorithms for exercise and posture classification. The final models were evaluated using the *F*_1_ score in a 10-fold cross-validation approach.

**Results:**

In total, 19 healthy adults with no history of LBP each completed at least one full session of exercises and postures. Random forest and XGBoost (extreme gradient boosting) models performed the best out of the initial set of 9 engineered feature models. The optimal hardware configuration was identified as a 3-sensor setup—lower back, left thigh, and right ankle sensors with acceleration, gyroscope, and magnetometer channels. The XGBoost model achieved the highest exercise (*F*_1_ score: mean 0.94, SD 0.03) and posture (*F*_1_ score: mean 0.90, SD 0.11) classification scores. The convolutional neural network achieved similar results with the same sensor locations, using only the accelerometer and gyroscope channels for exercise classification (*F*_1_ score: mean 0.94, SD 0.02) and the accelerometer channel alone for posture classification (*F*_1_ score: mean 0.88, SD 0.07).

**Conclusions:**

This study demonstrates the potential of a 3-sensor lower body wearable solution (eg, smart pants) that can identify exercises in multiple planes and proper sitting postures, which is suitable for the treatment of LBP. This technology has the potential to improve the effectiveness of LBP rehabilitation by facilitating quantitative feedback, early problem diagnosis, and possible remote monitoring.

## Introduction

Low back pain (LBP) is a prevalent condition that affects both physical and mental health [1,2]. Postural re-education and physiotherapy aiming to reduce disc derangement and strengthening exercises are often used to treat LBP [3-5]. Specifically, the McKenzie approach is based on a patient’s pain response to directional movements of the spine. The McKenzie approach has been proven to be effective and is commonly used by physiotherapists and other rehabilitation clinicians involved in the care of patients with LBP [2,3,6]. Research suggests that there is a positive correlation between adherence to rehabilitation programs (quantity and quality) and their ultimate success [4,7]. However, the quality of data (ie, derived from self-reported patient diaries) with respect to at-home rehabilitation program adherence can experience low rates of patient completion and biases [8]. A lack of a gold standard for measuring rehabilitation adherence has led to variability in the quality of measuring standards [8,9].

Image-based and wearable sensor systems have been used for assessing exercises and postures, applying methods developed within the broader field of human activity recognition (HAR) [10,11]. Image-based systems have many challenges (related to setup, line of sight, and computational requirements) that may limit their suitability for home-based rehabilitation assessment and posture monitoring [12]. Wearable sensors with inertial measurement units (IMUs) have been extensively used for HAR in diverse scenarios [13]. IMUs are easily embedded, compatible with multiple environments, and present fewer privacy concerns, suggesting a promising option for rehabilitation adherence and posture monitoring. Sensor placement, in the context of inertial sensors, has varied among HAR studies. Wang et al [14], O’Reilly et al [15], and Johnston et al [16] conducted reviews of wearable sensors used for the assessment of upper limb rehabilitation, lower limb rehabilitation, and posture, respectively. Recently, our group developed and validated a system to monitor home-based adherence to shoulder physiotherapy exercises (Smart Physiotherapy Adherence Recognition System [SPARS]) by using a single IMU (smartwatch) and state-of-the-art machine learning (ML) techniques [17-19]. However, LBP rehabilitation incorporates more complex movements than those found in the shoulder, which may not be adequately captured with a single IMU. As such, in developing a system to monitor LBP rehabilitation, it is important to determine the number of IMUs, their anatomical placement, and the data channels that best enable the classification of LBP rehabilitation exercises and posture.

The objective of this project was to develop and optimize a system to detect sitting posture and performance of LBP exercises comprising movement in multiple planes (flexion, extension, side glide, and rotation). It was hypothesized that inertial sensor time series data collected from a multi-IMU–based wearable device arrangement analyzed with ML will be able to successfully identify the performance of rehabilitation exercises and good sitting posture focused on reducing LBP.

## Methods

### Study Design and Participants

This study used a quantitative classification design to optimize a system that can detect sitting posture and performance of LBP exercises in a cohort of healthy participants. IMU data collected from multiple sensors were used to test and validate a range of ML models.

Healthy participants (from a limited cohort because of COVID-19 pandemic restrictions at that time) were recruited to participate in the study. Inclusion criteria were adult individuals with no prior history of LBP and a healthy BMI. Following informed consent, basic demographic data were collected (ie, age and sex) and study-specific ID numbers were assigned to each participant.

### Ethics Approval

Participants provided informed consent to participate in this study, and institutional research ethics board approval (research ethics board number: 3505; Sunnybrook Research Institute, Toronto, Ontario, Canada) was obtained.

### LBP Exercise and Posture Protocol

The McKenzie exercises represent a clearly defined, effective exercise set widely used by physiotherapists and other clinicians to treat LBP [2,6]. For this study, we selected a set of exercises that are used for the treatment of disc derangement. In total, 7 specific activities based on the McKenzie framework were identified for inclusion in the study protocol (1 static lying position and 6 dynamic lumbar spine exercises), as well as 3 postural positions. In addition, patients were recorded while performing various activities of daily living (ADL) such as walking, relaxed sitting, and standing. These ADL were collected so that models could be trained to not only differentiate between individual physiotherapy exercises but also to classify physiotherapy activities distinctly from typical daily activities. As such, these heterogeneous activities were all given the same ADL label to test the models’ ability to differentiate physiotherapy from other common activities as a general group. The exercise protocol incorporated flexion, extension, rotation, and side glide motions, as well as poor, good, and forced good sitting postures. The full list of exercises and postures is described in Multimedia Appendix 1.

Participants were trained to perform the exercises and postures under the direct supervision of a single researcher, following a protocol designed in collaboration with a McKenzie exercise–trained physiotherapist. Dynamic exercises were performed for 6 repetitions, and static exercises and sitting postures were performed for 30 to 60 seconds while wearing the multi-IMU sensor system. ADL activity data were collected for 3 to 5 minutes for each participant.

### Multisensor System

A wireless multi-IMU system was developed and used to collect inertial data during LBP rehabilitation for input to a classification model. The system comprised eight IMU devices (Metamotion C; Mbientlab) [20] placed in the following anatomical locations: (1) wrist, (2) left shoulder, (3) right shoulder, (4) upper back, (5) lower back, (6) left thigh, (7) right ankle, and (8) right ear. IMU locations are described in more detail in Figure S1 and the *IMU Locations* section in Multimedia Appendix 1.

The following five sensor data types, referred to here as *sensor channels*, were recorded from the IMU architecture (14 signal channels for each device, resulting in 112 channels):

1. Raw proper acceleration from the accelerometer (x, y, z), sampled at 25 Hz
2. Raw angular velocity data from the gyroscope (x, y, z), sampled at 25 Hz
3. Raw magnetic field strength from the magnetometer (x, y, z), sampled at 25 Hz
4. Quaternions from the on-board sensor fusion algorithm (w, x, y, z), sampled at 50 Hz
5. Pressure data from the barometer, sampled at 13 Hz

### Data Acquisition and Software

The SPARS software platform developed by our laboratory was extended to enable data acquisition from multiple IMUs [14-16]. To prevent sensor drift and accumulated magnetic interference, accelerometer, gyroscope, and magnetometer sensor channels were calibrated on a weekly basis according to the manufacturer’s instructions. IMUs were secured to each participant by using Velcro straps and adherent tabs, with 1 IMU integrated into a 3D printed earbud. Participants also donned a cap, a USB hub, and Bluetooth dongles. Data files were manually labeled by the supervising researcher, with the participant number and exercise class immediately after each exercise recorded. These labels served as the ground truth for subsequent classification tasks.

### Data Analysis

**Figure 1:**
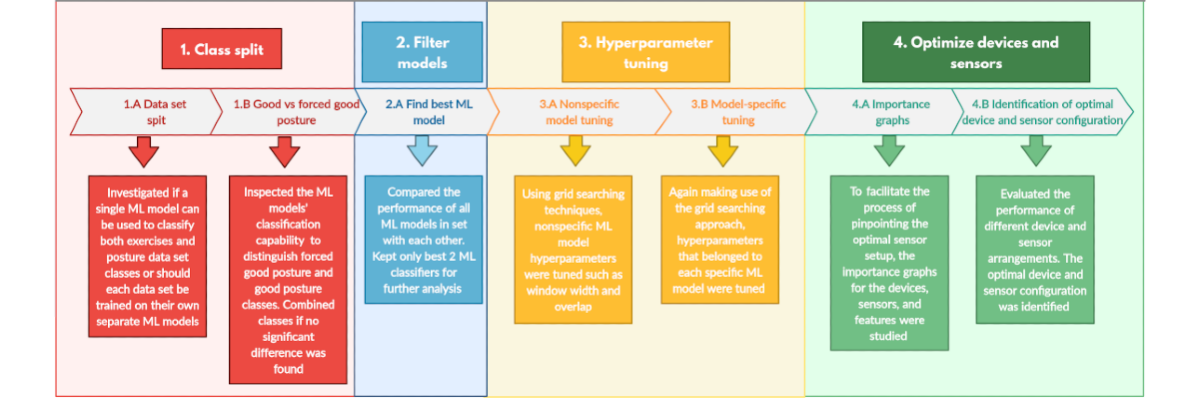
ML analysis flow. (1) Class split: to determine whether posture and exercise classification tasks require distinct classifiers and whether posture-forced good/posture good can be combined into a single class. (2) Filter models: identification of the 2 ML models with the highest performance from the classifier set. (3) Hyperparameter tuning: optimization of preprocessing parameters and model-specific hyperparameters. (4) Optimization of sensor channels and inertial measurement unit combinations: performing grid search over sensor channels and inertial measurement unit combinations, in addition to practical considerations for deployment. ML: machine learning.

The flow of the data analysis is outlined in Figure 1. The data collected using the SPARS-LBP system was used to determine the optimal placement of inertial sensors required to detect and classify LBP exercises and postures. This was accomplished by training a set of ML models to classify exercise data based on the full set of IMU sensor locations. A feature importance analysis was then performed to determine which IMUs and sensor channels contributed the most to model performance. Finally, a grid search was performed across a set of IMUs and channels, which contributed the most to the model performance. This was used to determine the optimal IMU locations, sensor channels, and model for a scalable SPARS-LBP system.

### Data Preprocessing

Raw accelerometer, gyroscope, magnetometer, and pressure data were taken directly from Mbientlab sensors for use in the training pipeline. These channels were also Kalman filtered and used to calculate the 4-axis quaternions channel with a proprietary Mbientlab algorithm. Filtering was applied to the quaternions channel by using the Bosch sensor fusion algorithm. This processing occurred during data acquisition.

The accelerometer, gyroscope, magnetometer, pressure, and quaternion channels were resampled to 25 Hz and segmented using a sliding window of a width of 5 seconds (125 samples), with 0 segment overlap. This segment length was chosen to be slightly longer than an average exercise repetition. The total number of segments after preprocessing was 7838 (5815/7838, 74.19% for exercise data, and 2024/7838, 25.82% for posture data).

### Feature Extraction and Scaling

Following segmentation, 23 statistical and time domain features (see the *Seglearn Engineered Features* section in Multimedia Appendix 1) were calculated for each channel of each time series segment, resulting in 2599 features. Segmentation and feature extraction were performed using the open-source Seglearn Python package [21]. Each feature was normalized to have 0 mean and unit variance before model training.

### Initial Models and Classification Task

In order to determine the optimal classification algorithm for use in subsequent experiments, 10 classifiers were initially considered: (1) decision tree, (2) random forest (RF), (3) XGBoost (XGB), (4) k-nearest neighbors, (5) support vector machine (SVM) trained with stochastic gradient descent, (6) linear discriminant analysis, (7) Gaussian naive Bayes, (8) SVM, (9) multilayer perceptron neural network, and (10) convolutional neural network (CNN).

Models 1 to 9 were trained on engineered features by using default settings from scikit-learn [22]. The CNN was trained directly on time series segments. The CNN comprised 3 convolutional layers with 128, 256, and 128 channels, respectively, followed by global average pooling, L2 normalization, and a fully connected layer. The CNN was trained for 100 epochs using the Adam optimizer, categorical cross-entropy loss, and a learning rate of 0.001. Initially, each model was trained with a 6-fold cross-validation approach on the entire data set, grouping folds based on participant. This ensured that recordings from the same participant were not present in both the train and test folds. A 6-fold cross-validation approach was chosen rather than a leave one participant out cross-validation approach because of the limited computational resources and time available to train 10 models. The class-weighted *F*_1_ score was used as the evaluation metric for all classification tasks.

Models were trained to perform three classification tasks: (1) classifying all exercises and postures (11-class output), (2) classifying only exercises (8-class output), and (3) classifying only posture (3-class output). The performance of the engineered features models (models 1-9) was evaluated. The 2 classifiers with the highest accuracies, lowest variance, and other supporting considerations (such as processing speed) were selected for further evaluation. In addition, the CNN model was considered for further optimization because of its previous success in classifying shoulder exercises [23].

### Feature Importance Evaluation

To determine the optimal combination of IMU locations and sensor channels for activity classification, the importance of engineered features was computed for the 2 selected pretrained engineered feature models. This was used to inform the selection of a subset of sensors and features for hyperparameter tuning. The following two methods of feature importance computation were explored: Gini importance (a measure of the number of branches learned from each feature in tree-based models [24]) and permutation feature importance (an approach where input features are randomly permuted and the change in model performance is measured [25]). The permutation approach is resilient to numerical feature inflation and training set dependence, which are found in Gini importance [24]. Features were then grouped by IMU location and sensor channel to determine the relative importance of each IMU and sensor channel.

### Hyperparameter Tuning and Sensor Selection

A grid search of model-specific and preprocessing hyperparameters was conducted, again using 6-fold cross-validation, grouping folds based on participant. The following preprocessing hyperparameters were included in the grid search because of their pronounced impact on time series features:

- Window width: Each exercise took approximately 5 seconds to complete, providing a maximum logical limit for the window. The lower limit was chosen as 0.5 seconds as smaller windows would not possess sufficient context.
- Window overlap: Overlap boundaries (overlap percentage between 2 consecutive window segments) were chosen to be 0%, representing no overlap at all and a maximum overlap of 60%. This parameter can also be considered a data augmentation parameter, where a higher overlap value results in more copies of similar segments.

The following model-specific hyperparameters were also considered. A full list of the model-specific hyperparameter space searched is available in Table S2 in Multimedia Appendix 1.

- RF: maximum features, minimum samples leaf, minimum samples split, and n estimators
- XGBoost: maximum depth, colsample bytree, gamma, learning rate, maximum depth, minimum child weight, and n estimators
- CNN: learning rate

IMU sensor channel combinations informed by the feature importance analysis were also included in the grid search. Owing to computational constraints, a smaller set of sensor channel combinations was chosen based on results from the engineered feature-based model grid search and used in a grid search for the CNN. This approach is similar to an embedded feature selection method. Finally, optimal configurations of the IMU locations and sensor channels were selected, considering supporting factors such as practicality and scalability as a future wearable system. The RF, XGBoost, and CNN models with optimized hyperparameters and input channels and IMU locations were retrained using a more rigorous 10-fold cross-validation approach, once again grouping folds based on the participant to prevent data leakage.

## Results

### Study Design and Participants

In total, 19 participants were recruited into the study, of whom 12 (63%) were male and 7 (37%) were female. Although specific height data for each patient were not recorded, all participants were within the healthy BMI range. Demographic data for the participants recruited for the study are displayed in Table 1.

**Table 1:** Demographic data collected for the participants recruited for the study (N=19).^a^

Characteristic		Participants
Age (years), mean (SD)		32 (12)
Body weight (kg), mean (SD)		76 (16)
**Sex, n (%)**		
	Male	12 (63)
	Female	7 (37)

^a^All participants had a healthy BMI and had no history of low back pain.

### Initial Models and Classification Task

Engineered feature models trained on a single classification task, combining exercise and posture activities using a 6-fold cross-validation strategy, did not perform as well as models separated into exercise classification (7 classes and ADL) and posture classification (3 classes). For 3-class posture classification, models were found to have a poor ability to distinguish between posture-forced good and good posture, suggesting little difference between the 2 postures. As a result, the good posture and posture-forced good groups were combined. The independent exercise (8 classes) and binary posture classifications were used for subsequent experiments.

The 10 models described in the methods were trained with default hyperparameters using a 6-fold cross-validation approach separately for exercise and posture data sets. The top 3 engineered feature models (shown in Table 2) with respect to average *F*_1_ score for exercise and posture classification were found to be RF, XGBoost, and SVM (0.85, 0.85, and 0.81 for exercise and 0.89, 0.89, and 0.88 for posture, respectively). However, the SVM was found to have a greater SD in the exercise set (0.12). As such, RF and XGBoost were identified as the models with the best performance for both exercise (0.85, SD 0.04) and posture (0.89, SD 0.07) classification problems.

**Table 2:** Initial averages and SDs of class-weighted F1 scores across 6-fold cross-validation for all 9 engineered feature-based models with default settings.^a^

Classifier	Exercise classification *F*_1_ score (weighted average), mean (SD)	Posture classification *F*_1_ score (weighted average), mean (SD)
Decision tree	0.76 (0.04)	0.81 (0.08)
Random forest	0.85 (0.04)^b^	0.89 (0.08)^b^
XGBoost	0.85 (0.04)^b^	0.89 (0.07)^b^
K-nearest neighbors	0.79 (0.04)	0.81 (0.11)
Stochastic gradient descent	0.83 (0.07)	0.76 (0.17)
Linear discriminant analysis	0.77 (0.11)	0.59 (0.09)
Gaussian naive Bayes	0.65 (0.09)	0.72 (0.14)
Support vector machine	0.81 (0.12)	0.88 (0.09)
Multilayer perceptron neural network	0.81 (0.14)	0.76 (0.18)

^a^Both exercise and posture classification tasks are shown, with all models using all sensor channels and inertial measurement unit locations as input.

^b^Top classifiers.

### Feature Importance Evaluation

**Figure 2:**
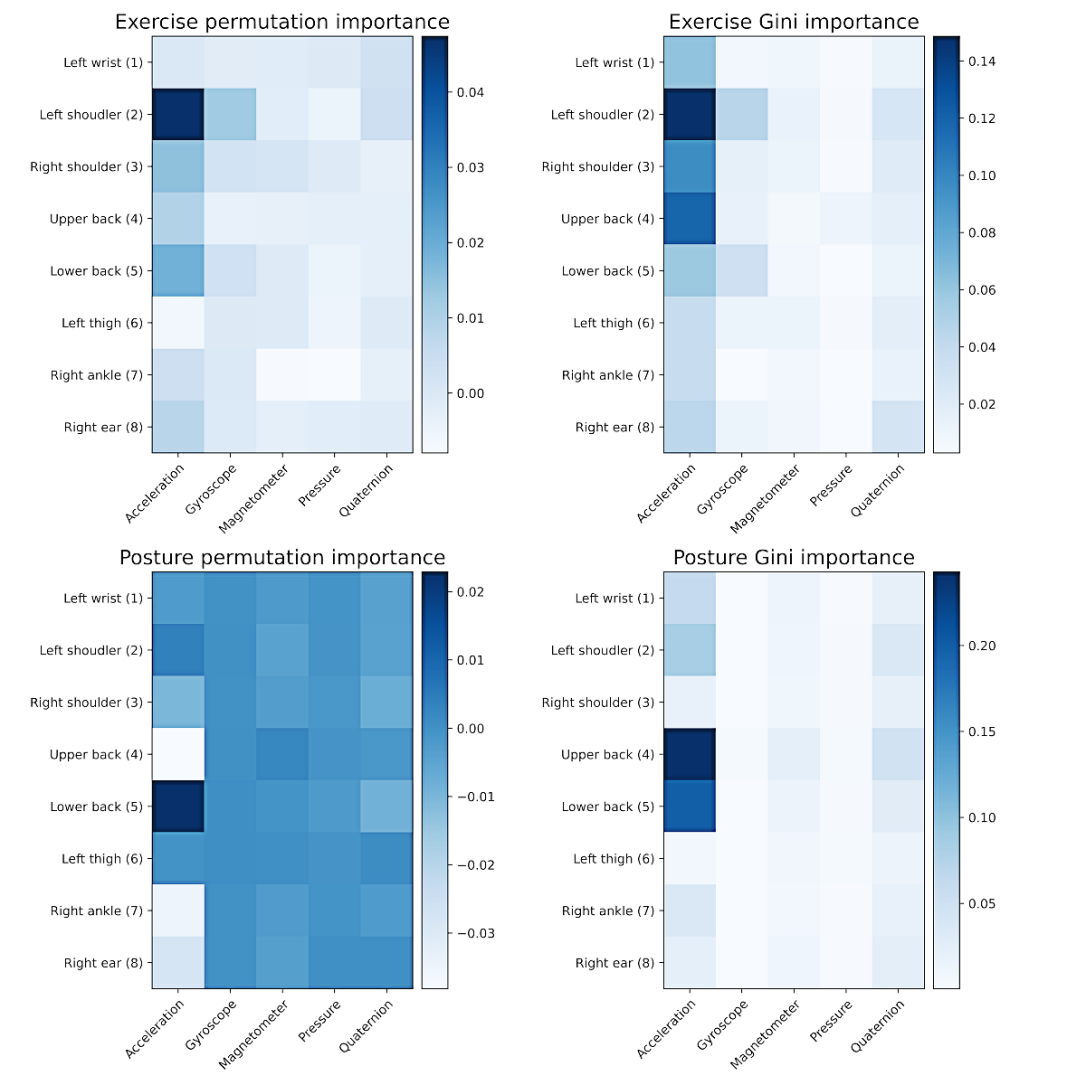
Average feature importance across 6-fold cross-validation for inertial measurement unit locations (y-axis) and sensor channels (x-axis). Larger values (darker blue) represent features of relatively high importance to the model. The permutation (left column) and Gini (right column) feature importances from the exercise random forest model are displayed in the top row, whereas the permutation and Gini importances for the posture random forest model are displayed in the bottom row. Importance values shown in the figure were computed by taking the sum across subchannels (eg, x, y, and z channels of acceleration) and engineered features for each subchannel. The resulting values represent the total feature importance for a given inertial measurement unit and channel combination (eg, “acceleration” and “upper back”), which were arranged in the grid shown here.

The XGBoost and RF models trained via 6-fold cross-validation to classify exercise and posture were used to compute the importance of input features. Inherent model importance (Gini importance and gain importance) and permutation importance for each of the input features were calculated (summarized in Figure 2 for the RF model). The importance of input features was then grouped based on IMU and sensor channel. Inherent model importance identified 5 (lower back), 6 (left thigh), and 7 (right ankle) as the most important devices; accelerometer and quaternions as the most important sensors; and mean, absolute energy, and absolute sum as the most important features. The permutation importance revealed that devices 7, 6, and 5 (reverse order compared with the Gini/Gain technique) had the highest importance; accelerometer and gyroscope had the highest importance among sensors; and minimum, absolute energy, and maximum had the top features contributing to classification performance. These findings were similar for the RF and XGBoost models.

### Hyperparameter Tuning and Sensor Selection

A grid search was conducted to identify the optimal model-specific hyperparameters and the optimal IMU sensor configuration. The results from the feature importance analysis were used to inform the selection of a subset of IMU sensor configurations for input to the hyperparameter grid search.

**Figure 3:**
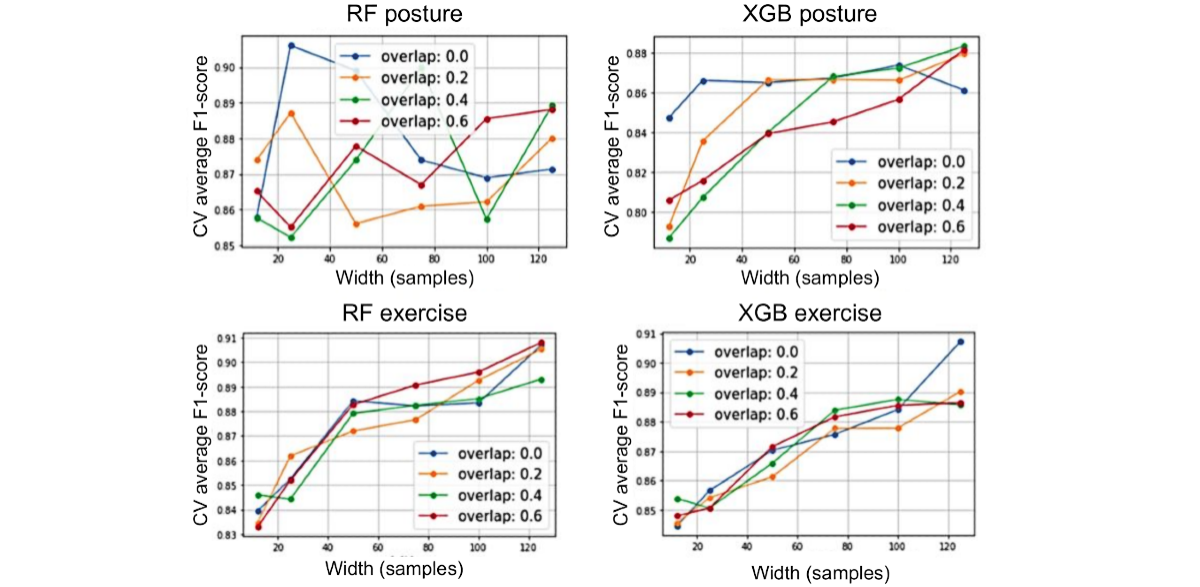
Hyperparameter grid search considering window width and overlap for the exercise (top row) and posture (bottom row) classification tasks for the RF (left) and XGBoost (right) models. Window width is shown to have a positive impact on performance for the exercise models, whereas no improvement is seen with overlap. Clear effectiveness is not demonstrated for the posture models with respect to window width or overlap. CV: cross-validation; RF: random forest; XGB: XGBoost.

The grid search results of the window width and window overlap parameters for both the exercise and posture classifications sets are displayed in Figure 3. Larger window width resulted in higher exercise classification performance in both engineered feature models and the CNN. Width had little impact on the performance of posture classification. Window overlap did not affect model performance for either classification task in the engineered feature models. A larger window overlap led to a small improvement in CNN exercise classification performance. Larger window widths were explored in the CNN grid search; however, the maximum window width was constrained by the length of recordings and, thus, was limited to 300 (12 seconds). On the basis of these findings, an optimal window width of 5 seconds and an overlap value of 0 were used for subsequent analyses with RF and XGBoost models, whereas a width of 300 and overlap of 50 samples (6% of the window) were used for the CNN.

Following the grid search conducted across model-specific hyperparameters, the performance of the engineered models did not show any significant improvements. *F*_1_ scores varied within +0.02 and –0.02 of the default hyperparameter results. As such, default RF- and XGBoost-specific hyperparameters were used for further analysis. The CNN performance was found to improve with a larger learning rate (0.66, SD 0.04 for learning rate=0.0001; 0.77, SD 0.050 for learning rate=0.01), with other hyperparameters held constant.

**Figure 4:**
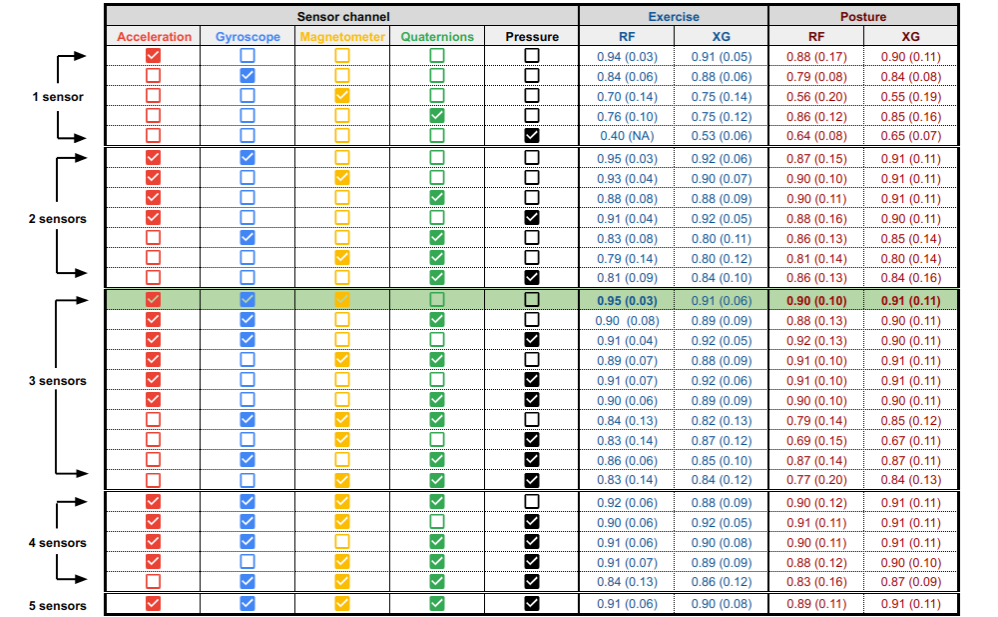
Results of the grid search across a set of sensor channel combinations for the RF and XGBoost models for exercise and posture classification. All IMUs were used for this test. Results are reported as the mean (SD) of the F1 score across 6-fold cross-validation for each sensor channel combination. The highlighted row represents the optimized sensor channels for both exercise and posture classification. RF: random forest; XG: XGBoost.

**Figure 5:**
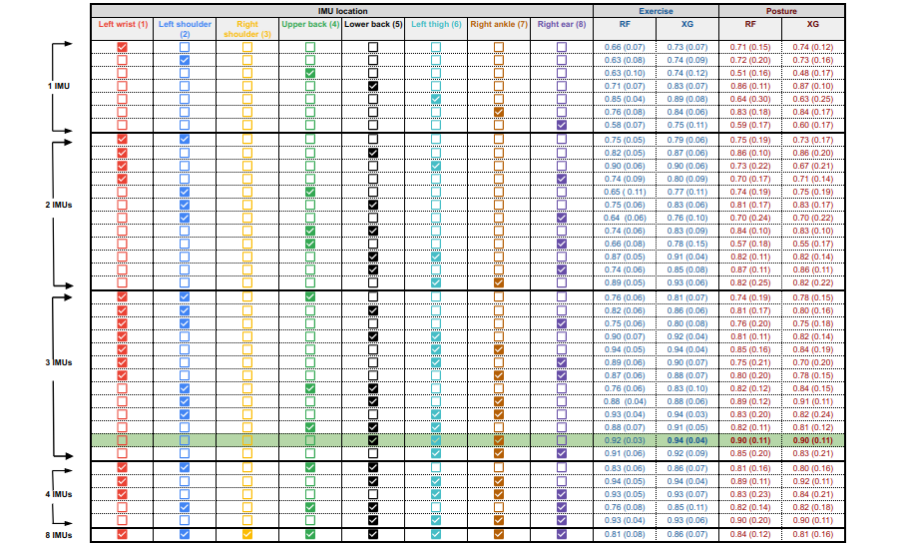
Results of the grid search across a set of IMU location combinations for RF and XGBoost models, classifying exercise and posture. All models were trained with the accelerometer, gyroscope, and magnetometer sensor channels. Results are reported as the mean (SD) of the F1 scores across 6-fold cross-validation for each IMU combination. The highlighted row represents the optimized sensor locations using 3 IMUs for both exercise and posture classification. Note that the bottom row containing all 8 IMUs is equivalent to the highlighted row in Figure 4. IMU: inertial measurement unit; RF: random forest; XG: XGBoost.

Finally, a set of IMU and sensor channel combinations, informed by the feature importance results, were included in the grid search for the RF and XGBoost models. The sensor channels evaluated using a 6-fold cross-validation approach, along with their corresponding performance scores, are displayed in Figure 4. The combination of accelerometer, gyroscope, and magnetometer sensors produced the best *F*_1_ scores of 0.95 (SD 0.03) and 0.91 (SD 0.11) for the exercise (RF model) and posture (RF model) data sets, respectively. The IMU combinations that were included in the grid search for the RF and XGBoost models are displayed in Figure 5. Using all available IMUs produced the highest performance. Limiting the number of sensors to 3 IMUs, the lower back (5), left thigh (6), and right ankle (7) locations yielded the best performance for both exercise (*F*_1_ score 0.94, SD 0.04) and e posture (*F*_1_ score 0.90, SD 0.11) using the XGBoost model. Confusion matrices for the final optimized IMU (5, 6, and 7) and sensor (accelerometer, gyroscope, and magnetometer) setup for RF and XGBoost models, trained with 10-fold cross-validation grouped based on participants, are provided in Figure S2 in Multimedia Appendix 1.

**Figure 6:**
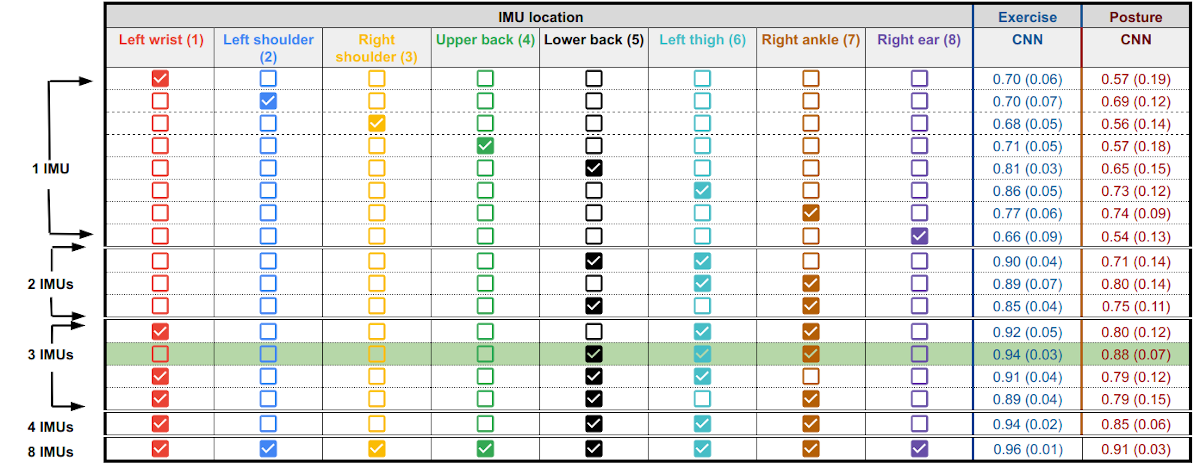
Results of the grid search across a subset of IMU locations and channel combinations for the CNN classifier. All models were trained with a segment width of 300, sampling rate of 25 Hz (total segment width of 12 seconds), overlap of 50, and learning rate of 0.001. These CNN grid search results used acceleration and gyroscope sensor channels for exercise classification and only the acceleration channel for posture classification. The reported F1 scores are the average (SD) across 6-fold cross-validation, stratified based on participant. CNN: convolutional neural network; IMU: inertial measurement unit.

Following the results showing that the low back, thigh, and ankle sensors performed optimally for exercise and posture classification with engineered feature models, a smaller subset of IMU combinations was tested with the CNN (Figure 6). A grid search was performed over the set of single IMUs in addition to the set of 2-IMU combinations, which could form a lower extremity garment (eg, pants or shorts). Furthermore, 3- and 4-IMU combinations that could form a lower extremity garment with a watch were examined. In the CNN grid search, the full set of IMUs provided the best performance (exercise *F*_1_ score 0.96, SD 0.01; posture *F*_1_ score 0.91, SD 0.03). The 3 best IMU systems for exercise were again 5, 6, and 7 (*F*_1_ score 0.94, SD 0.03 for exercise; *F*_1_ score 0.88, SD 0.07 for posture). The accelerometer and gyroscope proved to be the optimal sensor channel combination for the CNN for exercise classification, whereas only the accelerometer provided optimal performance for posture. Confusion matrices for the final optimized configurations for the CNN model, trained with 10-fold cross-validation, are also provided in Figure S2 in Multimedia Appendix 1.

## Discussion

### Principal Findings

This study demonstrated the ability of SPARS-LBP to successfully identify the performance of rehabilitation exercises and good posture based on sensor time series data collected from a multi-IMU–based wearable device arrangement. A large set of ML models were initially trained to classify exercise and posture activities by using the full set of sensors. RF and XGBoost were found to outperform 7 other engineered feature models during initial testing.

Mechanical pain of discogenic origin is perhaps the most common and treatable type of LBP. The application of McKenzie-based exercises requires extensive training by the clinicians to understand the pattern of disc derangement and directional preferences. Once the leg or buttock pain is centralized to the lumbar spine, the therapist can proceed with core strengthening exercises. We specifically chose McKenzie-based exercises as they do not address specific muscles and rather focus on the symptoms that originate from stimulation or deformation of the pain-sensitive structures by using mechanical means such as static positions or repeated end range movements.

In considering the practical deployment of the SPARS-LBP system, a reduction in the number of IMU and sensor signals was required. To this end, the relative importance of input features was computed and grouped by device and sensor for the RF model. This was performed using both the inherent Gini importance of each feature and the permutation importance. The Gini importance of a feature is determined by the number of splits in the tree originating from that feature. Therefore, it is a measure of a particular feature’s importance in the training data and can be misleading when the model overfits. For this reason, the permutation importance was also considered, as this can be computed on a held-out validation set using cross-validation [25]. The permutation importance is also limited by its tendency to assign low importance to highly correlated features. Owing to the drawbacks of each importance technique, the results of both methods were considered. This revealed that the thigh, ankle, wrist, lower back, and shoulder sensors along with the acceleration, gyroscope, and quaternion channels contributed the most to the performance of the model.

Ultimately, the ideal sensor and device combination was determined based on the grid search results and practical considerations for combining these sensors into a wearable system. An XGBoost model with 3 IMU devices placed at the lower back, thigh, and ankle, each recording accelerometer, gyroscope, and magnetometer data, was found to provide an optimal platform for exercise and posture classification. These 3 standard IMUs placed on the lower back, thigh, and ankle could ultimately be embedded into a single wearable garment (eg, pants).

The finding that the low back, thigh, and ankle sensors offered the optimal performance does not come as a surprise, as the low back exercises used in this study involve a variety of movements in the lower body. In particular, all the exercises and postures cause a displacement in the low back, which was found to have high importance in both the feature importance analysis and grid search. Interestingly, the sensors on the upper body (ear, shoulder, and wrist) offered relatively little improvement in performance. However, this result supports the future development of a single lower extremity garment.

We also found that the CNN had a comparable performance with the XGBoost model for exercise classification with these 3 device locations using accelerometer and gyroscope channels. The CNN was limited by the fact that longer segment widths of between 250 and 300 (10-12 seconds) were required for optimal exercise classification performance. This could present challenges in clinical settings where patients cannot perform exercises for more than a few seconds because of pain or other factors. However, for posture classification, the CNN’s best performance was achieved using just the acceleration sensor of all devices (*F*_1_ score 0.91, SD 0.03), and segment width did not seem to relate to performance. This is likely because of the stationary nature of the posture data, as the recording was performed once participants had already moved into position.

### Comparison With Prior Work

Although varied IMU setups have been studied for exercise classification (as in the study by Rodriguez et al [26]), none of them have explored simultaneous posture and exercise detection [27-31]. Some studies focused on lower limb rehabilitation exercises [15], whereas others focused only on postural classification [16]. Studies that focused on lower extremity rehabilitation found that the thigh, shin, and foot were useful sensor locations [15,32,33]. For posture experiments, sensor locations such as the lower back, upper back/sternum, feet, and thigh were used successfully [16]. These device locations described for exercise and posture studies coincide with our current findings, except for the upper back/sternum. Most studies that included the upper back/sternum as an IMU location used dynamic angle measurements to model their system in which they were used in relation to another device (usually a lower back IMU) or to an absolute starting position. These dynamic experiments also acquired data as participants repositioned themselves from a bad posture to a good posture, yielding time-varying fluctuations in the data. Using this approach, their algorithms would just need to learn the oscillations that occur in the upper back/sternum IMU when the posture changes to classify the data. By contrast, the postural data set collected in this study was static, where 1 good posture instance had the patient staying stationary for the whole period of the recording, with no time variation, limiting the importance of the upper back sensor (IMU 4) in the current algorithm.

### Strengths and Limitations

The performance of ML algorithms is generally dependent on the size of the available training data sets. Owing to COVID-19 pandemic restrictions, the recruitment of study participants and the resultant data set were limited. Participants were limited in the number of exercises and repetitions to avoid fatigue and prolonged session time. As such, the exercises used in this study represent a subset of all McKenzie exercise variations. Additional exercises may be explored to incorporate a larger exercise set, which will widen the scope and applicability of SPARS-LBP. This small data set resulted in greater interfold variability in cross-validation, particularly when larger segment widths were used, resulting in fewer training and validation samples. A second limitation is that feature selection was not used in the initial pipeline to select classifiers. This has the potential to penalize some classifiers (eg, k-nearest neighbors) and lead to overfitting. Third, this study demonstrated that the use of this technology is feasible, and the results are accurate; however, only healthy participants (without LBP and with healthy BMI) were included, and only correct execution of the LBP exercises was performed. Future work is needed to determine whether the optimized SPARS-LBP system can similarly classify exercises performed by those whose motion may be compromised and also consider the impact of age, sex, and BMI in relation to ML classification. A fourth limitation of the study was the restriction of monitoring devices to IMUs only. Testing a wider range of sensor technologies, such as electromyography or video data, could add to the robustness and accuracy of the classifications. However, there are challenges to the acquisition, synchronization, and analysis of multiple data streams, and the consideration of additional data sources is outside the scope of this study.

### Future Directions

As the use of wearable devices and artificial intelligence technology is expanding to facilitate web-based care, it is critical to explore the utility and accuracy of these devices in the musculoskeletal field. Validating the performance of the SPARS-LBP system with individuals prescribed McKenzie exercises for acute and chronic LBP is essential to see whether they generalize appropriately for these specific target populations. Essential to a clinical study is a simple IMU data acquisition system, such as a garment, incorporating the low back, thigh, and ankle IMU sensors. Similar to our ongoing work in the shoulder, this would allow elucidation of the relationship between participation and outcome (including functional assessment and patient-reported outcome measures). This would ultimately help guide future research into the effectiveness of physiotherapy programs and is key to understanding the relationships among exercise, posture, and clinical outcomes in individuals with LBP. App development may enable remote monitoring of participation/adherence by both patients and providers. This could allow for early identification of barriers to recovery while ensuring safe and effective management.

This work is also an important first step toward building effective tools to assess the quantity and quality of physiotherapy exercises. In particular, CNNs trained to classify physiotherapy exercises may be used to generate quantitative performance metrics based on generating embeddings for exercises performed in the clinic and at home by the same patient. Passing both recordings through the convolutional layers of the CNN, the distances between pairs of embeddings could then be used as a metric for the similarity between 2 exercises. Computing the similarity of a supervised exercise to an unsupervised exercise of the same class (performed at home) could give the patient and their clinician valuable feedback on the quality of unsupervised exercise performance.

### Conclusions

This study evaluated a large set of IMU devices (8) and sensors (5) during the performance of LBP exercises and sitting postures. The best performance was found using an optimized configuration of 3 IMUs (lower back, thigh, and ankle), with sensors limited to the accelerometer, gyroscope, and magnetometer. This device arrangement can be easily integrated into a wearable garment (pants) with a more efficient, simple, and clinically viable data acquisition system. No significant differences in performance of the 3 IMU systems were observed using the XGBoost, RF, and CNN models. This proof-of-concept study motivates further development of SPARS-LBP as a monitoring system that can help track participation and assist with the early identification of problems encountered in the performance of LBP exercise and correct posture, ultimately enhancing the effectiveness of at-home rehabilitation delivery.

## Appendix

Multimedia Appendix 1 Supplementary material containing detailed descriptions of the exercises, specific anatomic locations of the inertial measurement units (IMUs), the full list of model-specific hyperparameters included in the grid searches, and the confusion matrices of the optimized models.

## Abbreviations

ADL: activities of daily living
CNN: convolutional neural network
HAR: human activity recognition
IMU: inertial measurement unit
LBP: low back pain
ML: machine learning
RF: random forest
SPARS: Smart Physiotherapy Adherence Recognition System
SVM: support vector machine
XGB: XGBoost

## Data Availability

Data preprocessing and engineered feature extraction were performed with the Seglearn package [33]. The Python code used to create the convolutional neural network is also available [35]. The raw inertial measurement unit data can be found in the repository cited [36].
