# Supplementary material for: Detection of Low Back Physiotherapy Exercises With Inertial Sensors and Machine Learning: Algorithm Development and Validation
Source: JMIR Rehabil Assist Technol. 2022 Aug 23;9(3):e38689. doi: 10.2196/38689 (PMC9449825; doi:10.2196/38689)
Supplement: Multimedia Appendix 1 [file rehab_v9i3e38689_app1.docx]

**Multimedia Appendix 1**

*Supplementary material containing detailed descriptions of the exercises, specific anatomic locations of the inertial measurement unit (IMUs), the full list of model-specific hyperparameters included in the grid searches, and the confusion matrices of the optimized models.*

**1. Description of McKenzie Exercises**

**Postural exercises (PB, PG, PFG)**

In order to achieve a correct sitting posture, it is important to understand the impact of a poor sitting position on the spine, muscles, and discs. The slouch-overcorrect exercise is performed in sitting position. Sit in a slouched position in a chair or on a stool. Allow the lumber spine to rest on the ligaments in a fully flexed position and permit your head and chin to protrude forward (PB). To correct this position, move into an erect sitting posture with the lordosis at its maximum and the head held in retraction (PG). Use of a lumbar roll will help with the passive correction of the lumbar lordosis for prolonged sitting (PFG). Note postural positions and sustained (static) exercises were performed for 30-60 seconds.

**Sustained prone position (E1)**

Lying down in prone position with the arms alongside the trunk, rest your head on a pillow that is placed under your chest and head. In this position, the lumber spine automatically falls into some degree of lordosis.

**Dynamic extension in lying (E2)**

Place hands (palms down) underneath the shoulders as in a push-up position. Lift the top half of the body up by straightening the arms, while the pelvis is allowed to sag into the table. The top half of the body is then lowered and the exercise is repeated ten times. The level of extension is increased with the number of exercises. Most healthy individuals are able to obtain painfree full extension. Let the lumber spine relax into a sagged position. Note all dynamic exercises were performed with 10 repetitions.

**Dynamic side-glide in standing (E4)**

This exercise is performed for unilateral buttock or leg pain which does not respond to sagittal plane exercises. Stand and keep shoulders parallel to the ground. Place hand of the affected side (the side that is painful) on the ipsilateral hip and glide the pelvis to the opposite side without side bending your trunk. Stand for a minute or two in the overcorrection position.

**Dynamic extension in standing (E5)**

Stand with feet apart, place hands (thumbs on both sides of the spine) in the lumbar area. Extend your trunk backwards as far as possible, and then return to neutral standing.

**Dynamic rotation/flexion mobilization in flexion (E3, E7)**

Lie down in the supine position and keep shoulders firmly on the table. This will help rotation to occur in the lumbar spine. Flex both hips and knees to 90 degrees and rotate legs (E3 - both legs, E7 – one leg) to one side (therapist will decide on the side of rotation). Place your hand above the knee level to push knees toward the floor. This position is maintained for a few seconds.

**Dynamic flexion in lying (E6)**

While in lying supine position, bend knees fully and flex them toward the chest. Place hands at the knee level and apply overpressure to achieve maximum flexion. Knees are then released and feet are placed back on the mat.

**2.** **IMU Locations**


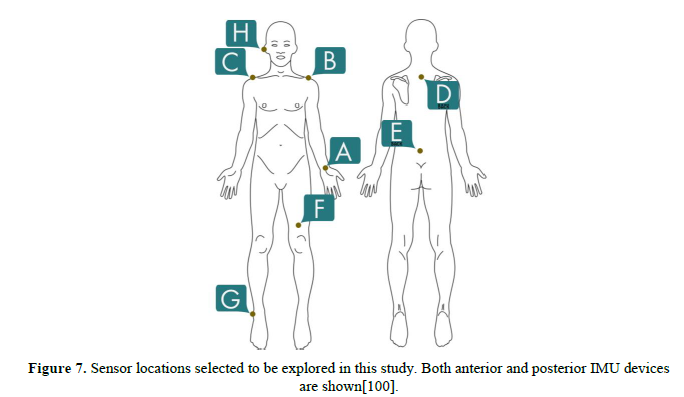
**Supplementary Figure S1**: IMU locations used for the study. Anatomic drawing taken from:

“Male Anatomy Front And Back Line Art - Openclipart.”

https://openclipart.org/detail/267821/male-anatomy-front-and-back-line-art (accessed Sep.

15, 2021).

Descriptions of IMU locations:

- A: Left Wrist: volarly, 3 cm proximal to the wrist joint.
- B: Left Shoulder: at the lateral edge of the acromion.
- C: Right Shoulder: at the lateral edge of the acromion
- D: Upper back: in the midline, at the level of the T2 spinous process,
- E: Lower Back: in the midline, at the level of the umbilicus
- F: Left Thigh: 2 cm proximal to the superior pole of the patella.
- G: Right Ankle: on the lateral malleolus
- H: Right Ear: Earbud is placed within the external auditory meatus

**3. Seglearn Engineered Features**

- Statistical features: mean, median, vector sum, absolute sum, absolute energy, standard deviation, variance, mean absolute deviation, variation, min, max, skewness, kurtosis.
- Time domain features: mean derivative, mean absolute derivative, mean spectral energy, mean crossings, mean absolute value, zero crossings, slope sign changes, waveform length, root mean square, Willison amplitude.

**4. Model Specific Hyperparameter Grid Search Space**

**Supplementary Table S1:** Full list of model-specific hyperparameters which were used in the grid search. The random forest hyperparameter names correspond to the keyword arguments in Scikit-Learn’s RandomForestClassifier class. Hyperparameter names for the XGBoost model refer to keyword arguments in XGBoost’s XGBoostClassifier class.

| **Hyperparameters** | **Random Forest** | **XGBoost** | **CNN** |
| --- | --- | --- | --- |
| learning_rate | N/A | 0.05, 0.10, 0.20, 0.30 | (0.1, 0.01, 0.001, 0.0001). |
| *n_estimators* | 100, 480, 860, 1240, 1620, 2000 | 100, 200, 500 | N/A |
| *max_features* | “auto”, “sqrt” | N/A | N/A |
| *max_depth* | 10, 57, 105, 152, 200 | 3, 7, 15 | N/A |
| *min_samples_split* | 2, 10, 20, 30 | N/A | N/A |
| *min_samples_leaf* | 1, 2, 4, 8, 12 | N/A | N/A |
| *bootstrap* | True, False | N/A | N/A |
| min_child_weight | N/A | 1, 4, 7 | N/A |
| subsample | N/A | 0.5, 0.7 | N/A |
| gamma | N/A | 0.0, 0.2 , 0.4 | N/A |
| colsample_bytree | N/A | 0.3, 0.5, 0.7 | N/A |
| objective | N/A | 'reg:squarederror' | N/A |



**Supplementary Figure S2.** Confusion Matrices representing the performance of the RF (top row), XGB (middle row), and CNN (bottom row) models for the optimized 3 IMU system (E,F,G) with a 10 fold cross validation for exercise classification (left) and posture classification (right). Confusion matrices show the proportion of samples from each class (True label) that were classified into each class (predicted label). Darker blues along the diagonal represent higher classification performance, while shades of blue on the off-diagonal show incorrect classification. E1-E7 and ADL represent the LBP exercises. PB and PG represent poor and good sitting posture, respectively.
